# The effect of purification of Ga-68-labeled exendin on in vivo distribution

**DOI:** 10.1186/s13550-016-0221-8

**Published:** 2016-08-12

**Authors:** Maarten Brom, Gerben M. Franssen, Lieke Joosten, Martin Gotthardt, Otto C. Boerman

**Affiliations:** Department of Radiology and Nuclear Medicine, Radboud University Nijmegen Medical Centre, PO Box 9101, 6500 HB Nijmegen, The Netherlands

**Keywords:** ^68^Ga, ^68^Ga-hydroxide, Purification, Peptides, Exendin

## Abstract

**Background:**

Ga-labeled radiotracers are increasingly used for PET imaging. During the labeling procedure, formation of ^68^Ga-colloid may occur. Upon i.v. injection, ^68^Ga-colloid will accumulate rapidly in the liver, spleen, and bone marrow, resulting in reduced target-to-background ratios. In this study, we applied a thin layer chromatography (TLC) method to measure colloid content and we studied the effect of the purification method on the in vivo characteristics of ^68^Ga-labeled DOTA-exendin-3.

DOTA-exendin-3 was labeled with ^68^Ga, and the colloid content was measured by TLC on silica gel ITLC with two mobile phases. The labeling mixture was purified by gel filtration on a 5-ml G25M column, by reversed-phase high-performance liquid chromatography (RP-HPLC) using a C_8_ column or by solid phase extraction (SPE) on an HLB cartridge. The in vivo characteristics of the preparations were determined in BALB/c nude mice, and PET images were acquired 1 h p.i. using a microPET scanner. In these studies, unpurified ^68^Ga-DOTA-exendin-3 and ^111^In-DOTA-exendin-3 were used as a reference.

**Results:**

The colloid content of ^111^In-DOTA-exendin-3 and unpurified, gel filtration, RP-HPLC- and SPE-purified ^68^Ga-DOTA exendin-3 was <3, 7, 9, <3, and <3 %, respectively. Unpurified ^68^Ga-DOTA exendin-3 showed high liver and spleen uptake. Gel filtration partly removed ^68^Ga-colloid from the preparation, resulting in moderate liver and spleen SPE-purified ^68^Ga-DOTA exendin-3 showed very low liver and spleen uptake, that was similar to that of RP-HPLC purified ^68^Ga-DOTA exendin-3.

**Conclusions:**

We showed that the colloid content can be measured by TLC and that solid phase extraction and HPLC completely remove ^68^Ga-colloid from ^68^Ga-labeled tracer preparations, resulting in very low liver and spleen uptake. This study clearly shows the importance of removal of ^68^Ga-colloid from preparations.

## Background

^68^Ga-labeled peptides are increasingly used for positron emission tomography (PET), since ^68^Ga is a readily available PET radionuclide. Because ^68^Ga is a generator-produced positron emitter, it is widely available and relatively cheap. PET imaging is advantageous over conventional scintigraphy and SPECT because of its excellent sensitivity in combination with its superior spatial resolution. A recent study in patients with neuro-endocrine tumors (NET) showed that PET imaging with the somatostatin analog ^68^Ga-DOTA-TOC was more sensitive for detecting NET lesions than conventional somatostatin receptor scintigraphy with ^111^In-octreotide [1]. Moreover, ^68^Ga-labeling of peptides conjugated with a chelator (e.g., DOTA, NOTA) is a fast and efficient one-step reaction. Because of all these characteristics, there is an increasing interest in the application of ^68^Ga-labeled peptides.

It has been shown that for efficient receptor targeting, low peptide doses should be administered, since higher peptide doses could lead to receptor saturation and reduced uptake in the target tissue [2–5], especially in preclinical imaging studies. In rodents, relatively high activity doses (3–10 MBq) have to be administered to acquire PET images with adequate image quality. Therefore, ^68^Ga-labeled peptides with a high specific activity should be produced to administer high activity doses at a low amount of peptide. However, when producing ^68^Ga-labeled compounds with a high specific activity, the formation of insoluble ^68^Ga-species, such as ^68^Ga(OH)_3_ may occur. These insoluble ^68^Ga-species, generally referred to as “^68^Ga-colloid” will accumulate in the liver, spleen, and bone marrow. Indeed, previous studies showed enhanced uptake in liver and spleen of ^68^Ga-labeled tracers as compared to the ^111^In-labeled compounds [3, 6, 7]. This enhanced tracer uptake in liver and spleen might result in decreased target-to-background ratios. We previously showed that insoluble ^68^Ga-species can be removed from the labeling reaction of ^68^Ga-labeled DOTA-exendin-3, a tracer targeting the glucagon-like peptide-1 receptor (GLP-1R), using (preparative) reversed-phase high-performance liquid chromatography (RP-HPLC) [3]. However, this purification method is time consuming and the solution of purified ^68^Ga-labeled peptide is diluted, making post-purification concentration necessary. Due to the short half-life of ^68^Ga (68 min), this method is not convenient in clinical practice. Moreover, purification with RP-HPLC requires expensive equipment. Solid phase extraction is an alternative method for purification of radiolabeled compounds and is a fast, simple, and cheap purification method that is now routinely used for ^68^Ga-tracer purification [8, 9].

In this study, we examined the effect of the purification method on the in vivo characteristics of ^68^Ga-DOTA-exendin-3 in BALB/c nude mice. ^68^Ga-DOTA-exendin-3 was purified by RP-HPLC, gel filtration, or solid phase extraction. Unpurified ^68^Ga-DOTA-exendin-3 and ^111^In-DOTA-exendin-3 were used as a reference in this study. In addition, we describe a quality control method, based on instant thin layer chromatography (ITLC), to determine the colloid content of the ^68^Ga-labeled tracer [10].

## Methods

### Peptides and radionuclides

[Lys^40^(DOTA)]exendin-3 (DOTA-exendin-3) was purchased from Peptide Specialty Laboratories (Heidelberg, Germany). In this compound, DOTA (1,4,7,10-tetraazacyclododecane-1,4,7,10-tetraacetic acid) is conjugated to the ε-amino group of the lysine at position 40 (K40) and the C-terminal carboxyl group is amidated [3]. ^68^GaCl_3_ was eluted from a TiO_2_-based 1110 MBq ^68^Ge/^68^Ga generator (IGG100, Eckert and Ziegler, Berlin, Germany) with 0.1 N Ultrapure HCl (J.T. Baker, Deventer, The Netherlands). ^111^InCl_3_ was obtained from Covidien (Petten, The Netherlands).

### Radiolabeling

DOTA-exendin-3 was labeled with ^68^Ga and ^111^In as previously described [3]. Briefly, 120 MBq ^68^Ga in 1000 μl Ultrapure 0.1 N HCl was added to 10 μg DOTA-exendin-3 in 120 μl 2.5 M HEPES (4-(2-hydroxyethyl)-1-piperazineethanesulfonic acid, Sigma Aldrich, St. Louis, MO, USA). After 20-min incubation at 95 °C, EDTA was added to a final concentration of 5 mM and the reaction mixture was incubated at room temperature for another 5 min. Subsequently, 10 % Tween-80 (Sigma Aldrich, St. Louis, MO, USA) was added to a final concentration of 0.1 % to prevent sticking of the radiolabeled peptide to the vessel wall and quality control was performed as described below.

DOTA-exendin-3 was labeled with ^111^In by adding 10 MBq ^111^InCl_3_ to 1 μg peptide in 0.1 M 2-(*N*-morpholino)ethanesulfonic acid (MES), pH 5.5, under similar conditions as described above.

### Quality control

Quality control was performed using RP-HPLC on a C_18_ reversed-phase column (Zorbax Rx-C18; 4.6 mm × 25 cm; Agilent Technologies, Palo Alto, CA, USA) and instant thin layer chromatography (ITLC). The column was eluted with mixture of water containing 0.1 % trifluoroacetic acid (TFA) and acetonitrile with a linear gradient from 3 to 100 % acetonitrile in 10 min (flow rate 1 ml/min). ITLC was performed on silica gel ITLC (Pall Corporation Life Sciences, New York, NY, USA). Two mobile phases were used: 0.1 M EDTA in 0.25 M NH_4_Ac, pH 5.5 (*R*_f_ = 0: ^68^Ga-labeled exendin and ^68^Ga-colloid, *R*_f_ = 1: ^68^Ga-EDTA) and 1.25 M NH_4_Ac, pH 5.5: dimethylformamide (DMF) (1:1) (*R*_f_ = 0: ^68^Ga-colloid, *R*_f_ = 1: ^68^Ga-DOTA-exendin-3 and ^68^Ga-EDTA).

### Validation of the quality control by ITLC for the detection of ^68^Ga-colloid

^68^Ga-colloid was prepared by adding 1250 μl 2.5 M HEPES to 500 μl ^68^GaCl_3_ in 0.1 M HCl. The final pH of this mixture was approximately 6. The mixture was incubated at 95 °C for 15 min, and EDTA was added to a final concentration of 5 mM. The amount of ^68^Ga-colloid was determined by TLC with 0.1 M EDTA in 0.25 M NH_4_Ac, pH 5.5 as a mobile phase (*R*_f_ = 0: ^68^Ga-colloid, *R*_f_ = 1 ^68^Ga-EDTA) and 1.25 M NH_4_Ac, pH 5.5: DMF (1:1) as a mobile phase (*R*_f_ = 0: ^68^Ga-colloid, *R*_f_ = 1: ^68^Ga-EDTA). The reaction mixture was applied on a disposable PD-10 desalting column, containing Sephadex^TM^ G-25 medium (GE Life Sciences, Diegem, Belgium) and was eluted with 6 ml phosphate-buffered saline (PBS) containing 5 mM EDTA. The fraction containing the majority of the radioactivity was collected (from 3–4 ml), representing ^68^Ga-colloid, and quality control was performed by ITLC as described above.

^68^Ga-DOTA-exendin-3 was purified by RP-HPLC as described below, and various amounts of the ^68^Ga-colloid were added to obtain final ^68^Ga-colloid concentrations of 1, 2, 3, 4, 5, 10, 20, 40, and 80 % (*n* = 4). The amount of ^68^Ga-colloid was determined by ITLC with 1.25 M NH_4_Ac, pH 5.5: DMF (1:1) as the mobile phase (*R*_f_ = 0: ^68^Ga-colloid, *R*_f_ = 1: ^68^Ga-DOTA-exendin-3 and ^68^Ga-EDTA). RP-HPLC-purified ^68^Ga-DOTA-exendin-3, ^68^Ga-EDTA, and ^68^Ga-colloid were used as controls. ITLC strips were exposed to an imaging plate (Fuji Film BAS-SR 2025, Raytest, Straubenhardt, Germany) for 1 min. Images were acquired with a radioluminography laser imager (Fuji Film BAS 1800 II system, Raytest, Straubenhardt, Germany) and analyzed with Aida Image Analyzer software (Raytest). Correlation between the measured ^68^Ga-colloid fraction and the added ^68^Ga-colloid content was determined by linear regression using GraphPad Prism 5. The detection limit of the ITLC method was defined by the Y-intercept as determined by linear regression analysis.

### RP-HPLC purification of ^68^Ga-DOTA-exendin-3

After radiolabeling, the reaction mixture was purified by HPLC, using a C_8_ reversed-phase column (Zorbax eclipse XDB C_8_ 4.6 mm × 150 mm, 5 μm, Agilent Technologies). The column was eluted with water containing 0.1 % TFA (0–5 min), 40 % ethanol (5–10 min) followed by a linear gradient from 40 to 90 % ethanol in 5 min (flow rate 1 ml/min). The fractions containing ^68^Ga-DOTA-exendin-3 (retention time 14–15 min) were collected and diluted with PBS containing 0.5 % bovine serum albumin (BSA) to a final ethanol concentration of less than 10 % before injection into mice (injection volume 0.2 ml). The radiochemical purity of the purified ^68^Ga-labeled DOTA-exendin-3 preparations was determined using ITLC as described above.

### Purification of ^68^Ga-DOTA-exendin-3 by gel filtration

Purification by gel filtration was performed on disposable PD-10 desalting columns, containing Sephadex^TM^ G-25 medium (GE Life Sciences, Diegem, Belgium). The column was preconditioned by eluting with 10 ml PBS containing 0.5 % (*v*/*w*) BSA, and the reaction mixture was loaded onto the column. The column was eluted with PBS-BSA (0.5 %), and 0.5 ml fractions were collected. The majority of the radioactivity, representing ^68^Ga-DOTA-exendin-3, was collected in fractions 5 and 6 (2-3 ml). The radiochemical purity of the tracer in the fractions was analyzed by RP-HPLC and ITLC as described above.

### Purification of ^68^Ga-DOTA-exendin-3 by solid phase extraction

Solid phase extraction was performed using a hydrophilic-lipophilic balance (HLB) reversed-phase sorbent cartridge (Waters Oasis^©^, Milford, MA, USA). The cartridge was activated by elution with 1 ml ethanol, the residual ethanol was removed with 1 ml water (Versol, Lyon, France), and the column was conditioned with 1 ml 0.1 N HCl:2.5 M HEPES (8:1, similar to the reaction mixture). The reaction mixture was loaded, and ^68^Ga-EDTA was washed from the column with 2 ml 0.1 N HCl:2.5 M HEPES (8:1). After removal of the residual HCl-HEPES mixture with 1 ml water, ^68^Ga-DOTA-exendin-3 was eluted with 200 μl ethanol. The radiochemical purity of the tracer in the fractions was analyzed by RP-HPLC and ITLC as described above. Before injection into mice, the eluate containing ^68^Ga-DOTA-exendin-3 was diluted with PBS containing 0.5 % BSA to a final ethanol concentration of less than 10 %.

### Biodistribution studies

Animal experiments were performed after approval of the local ethical committee (RUDEC) for animal experiments. The biodistribution of unpurified, gel filtration-, RP-HPLC-, and SPE-purified ^68^Ga-DOTA-exendin-3 was determined in BALB/c nude mice. Mice (*n* = 5) were injected intravenously with 3 MBq ^68^Ga-labeled exendin-3 at a peptide dose of 0.3 μg (60 pmol). As a control, another group of mice received 370 kBq ^111^In-DTPA-exendin-3 (60 pmol). The mice were euthanized 1 h post-injection by CO_2_/O_2_ suffocation, a blood sample was taken, and samples of relevant tissues were dissected, weighed, and counted.

### MicroPET

Mice were injected intravenously with 3 MBq (0.3 μg) unpurified, gel filtratrion, RP-HPLC- or SPE-purified ^68^Ga-DOTA-exendin-3. Mice were euthanized 1 h p.i. by CO_2_/O_2_ suffocation and PET images were acquired during 45 min using a small-animal PET/CT scanner (Inveon™; Preclinical Solutions, Siemens Medical Solutions USA, Inc., Knoxville, TN, USA). Images were reconstructed by OSEM3D/MAP reconstruction with the following parameters: 256 × 256 matrix, 2 OSEM3D iterations, 18 MAP iterations, and a resolution of 0.075 mm uniform variance. CT images were acquired for anatomical correlation directly after PET imaging (spatial resolution 113 μm, 80 kV, 500 μA, exposure time 300 ms).

### Statistical analysis

All mean values are expressed as mean ± standard deviation (SD). Statistical analysis was performed using unpaired two-tailed *t* test using GraphPad Prism (version 5). In order to determine whether there was a overall difference in blood and kidney accumulation of the various preparations, a one-way ANOVA was performed. The level of significance was set at *p* < 0.05.

## Results

### Radiolabeling

DOTA-exendin-3 could be labeled with ^68^Ga with a specific activity of 110 GBq/μmol (at time of synthesis) with a radiochemical purity >90 % (depending on the purification method) after purification. The colloid content of ^111^In-DOTA-exendin-3 and unpurified, gel filtration, RP-HPLC- and SPE-purified ^68^Ga-DOTA-exendin-3 was <3, 7, 9, <3, and <3 %, respectively (Table 1). Of note, the initial colloid content of the labeling mixture varies, which explains the higher colloid content of ^68^Ga-DOTA-exendin-3 after gel filtration. After purification, the percentage of unincorporated ^68^Ga was <3 % for all preparations as determined by RP-HPLC.

**Table 1:** ^68^Ga-colloid content determined by ITLC, liver, and spleen uptake of unpurified, gel filtration, RP-HPLC- and SPE-purified ^68^Ga-DOTA-exendin-3 and ^111^In-DOTA-exendin-3 in BALB/c nude mice (*n* = 5 per group)

	^68^Ga-colloid content (%)	Liver uptake (% ID/g)	Spleen uptake (% ID/g)
^111^In-DOTA-exendin-3	<3	0.7 ± 0.1	0.3 ± 0.1
^68^Ga-DOTA-exendin-3	7	6.1 ± 1.0	4.5 ± 0.7
Gel filtration purified ^68^Ga-DOTA-exendin-3	9	3.0 ± 0.3	1.4 ± 0.3
Solid phase extraction purified ^68^Ga-DOTA-exendin-3	<3	0.8 ± 0.0	0.5 ± 0.1
RP-HPLC purified ^68^Ga-DOTA-exendin-3	<3	0.6 ± 0.1	0.4 ± 0.1

### Validation of TLC method

**Fig. 1:**
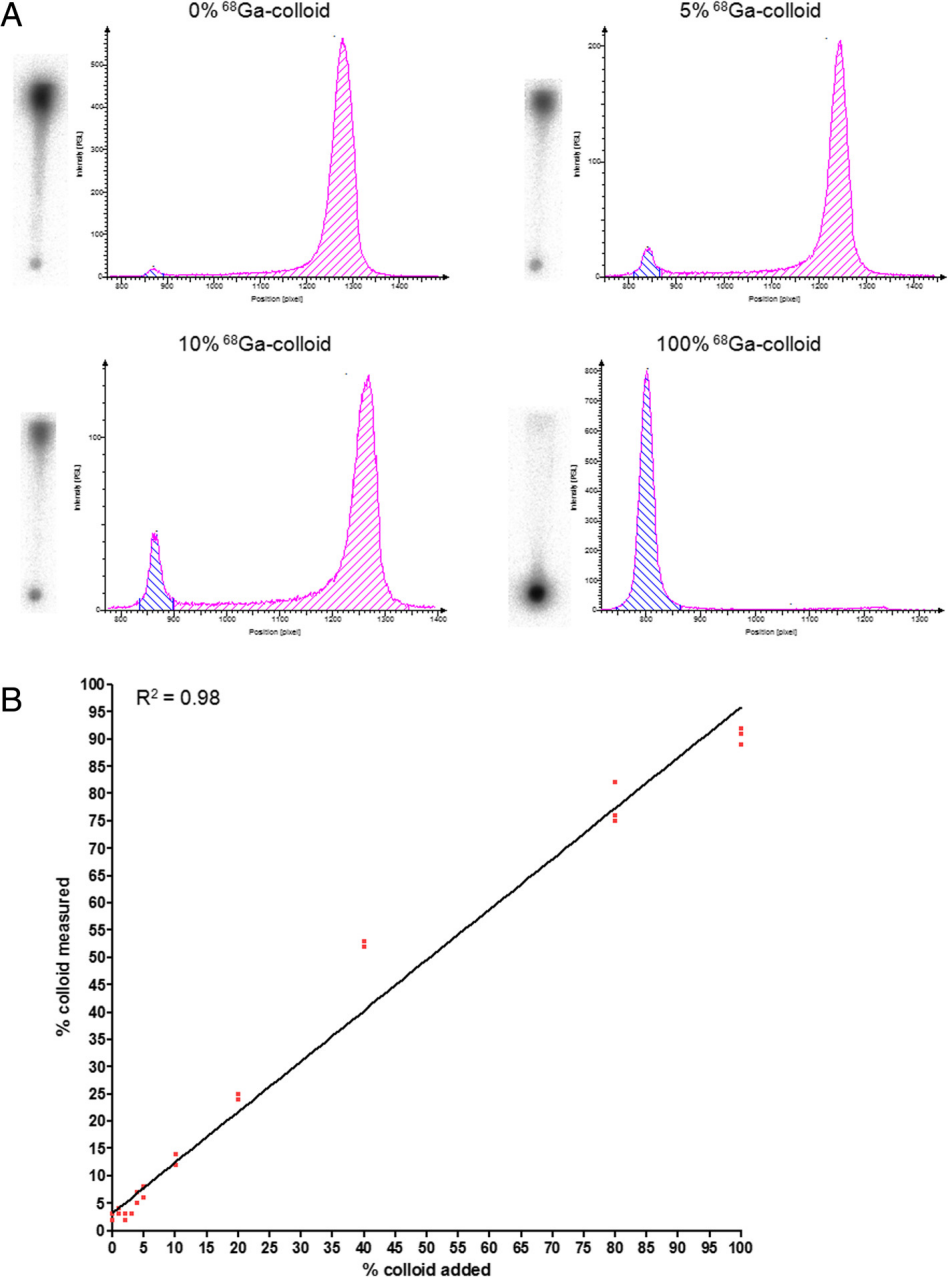
ITLC method for determination of the ^68^Ga-colloid content. **a** TLC images and profiles of ^68^Ga-DOTA-exendin-3 with various amounts of ^68^Ga-colloid (typical examples). **b** Correlation between the amount of ^68^Ga-colloid added and the measured colloid content as determined by TLC

The results of the validation of the TLC method to determine the colloid content in preparations of ^68^Ga-DOTA-exendin-3 are shown in Fig. 1. The colloid content measured by TLC correlated linearly with the amount of colloid present in the reaction mixtures (*R*^2^ = 0.98). The detection limit of the TLC method was a ^68^Ga-colloid content of 3.2 ± 0.9 %, as determined by the Y-intercept of the trend line.

### Biodistribution studies

**Fig. 2:**
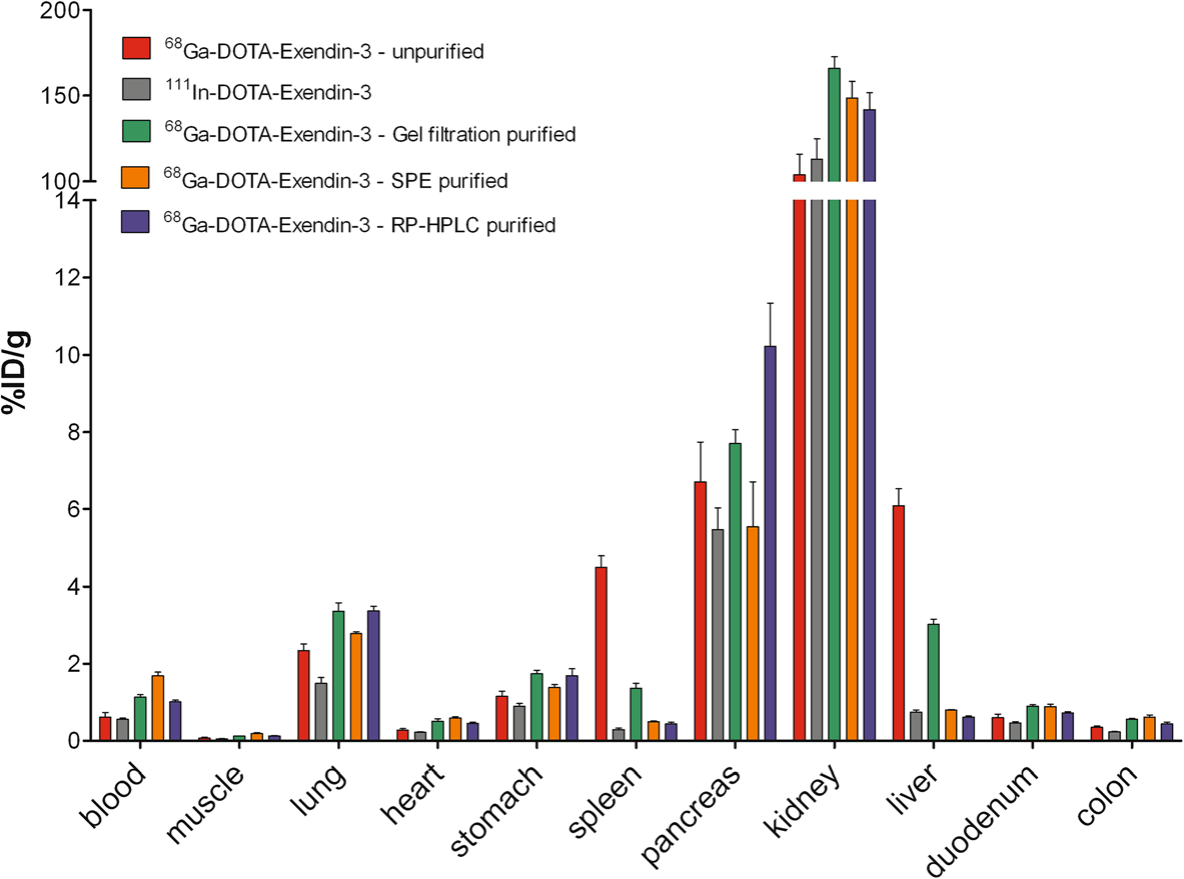
Biodistribution of unpurified, gel filtration, RP-HPLC and SPE-purified ^68^Ga-DOTA-exendin-3 in BALB/c nude mice (*n* = 5 per group). ^111^In-DOTA-exendin-3 was used as a reference. Values are expressed as percentage of injected dose per gram of tissue (%ID/g) ± SD. Mice were dissected 1 h p.i

The results of the biodistribution studies are summarized in Fig. 2. ^111^In-DOTA-exendin-3 showed very low accumulation in the liver and spleen: 0.7 ± 0.1 and 0.3 ± 0.1 %ID/g, respectively, representing the liver and spleen uptake of a preparation without colloid. High uptake in the liver and spleen (6.1 ± 1.0 and 4.5 ± 0.7 %ID, respectively) was observed when unpurified ^68^Ga-DOTA-exendin-3 was injected. Gel filtration partially removed ^68^Ga-hydroxide, resulting in intermediate uptake in the liver and spleen: 3.0 ± 0.3 and 1.4 ± 0.3 %ID/g, respectively). Uptake in the liver and spleen of SPE-purified (0.8 ± 0.0 %ID/g and 0.5 ± 0.1 %ID/g, respectively) and RP-HPLC-purified tracer (0.6 ± 0.1 and 0.4 ± 0.1 %ID/g, respectively) was very low and similar to liver and spleen uptake of ^111^In-DOTA-exendin-3, indicating very efficient removal of ^68^Ga-colloid with these purification methods.

The uptake in the pancreas was similar for all compounds, except of RP-HPLC purified ^68^Ga-DOTA-exendin-3, that had a significantly higher pancreatic uptake (10.2 ± 2.5 %ID/g, *p* < 0.05). A significant difference in radioactivity concentration in the blood and kidneys was found between all groups (*p* < 0.0001 and *p* = 0.0035 for blood and kidneys, respectively), probably caused by differences in the colloid content of the various preparations.

### MicroPET

**Fig. 3:**
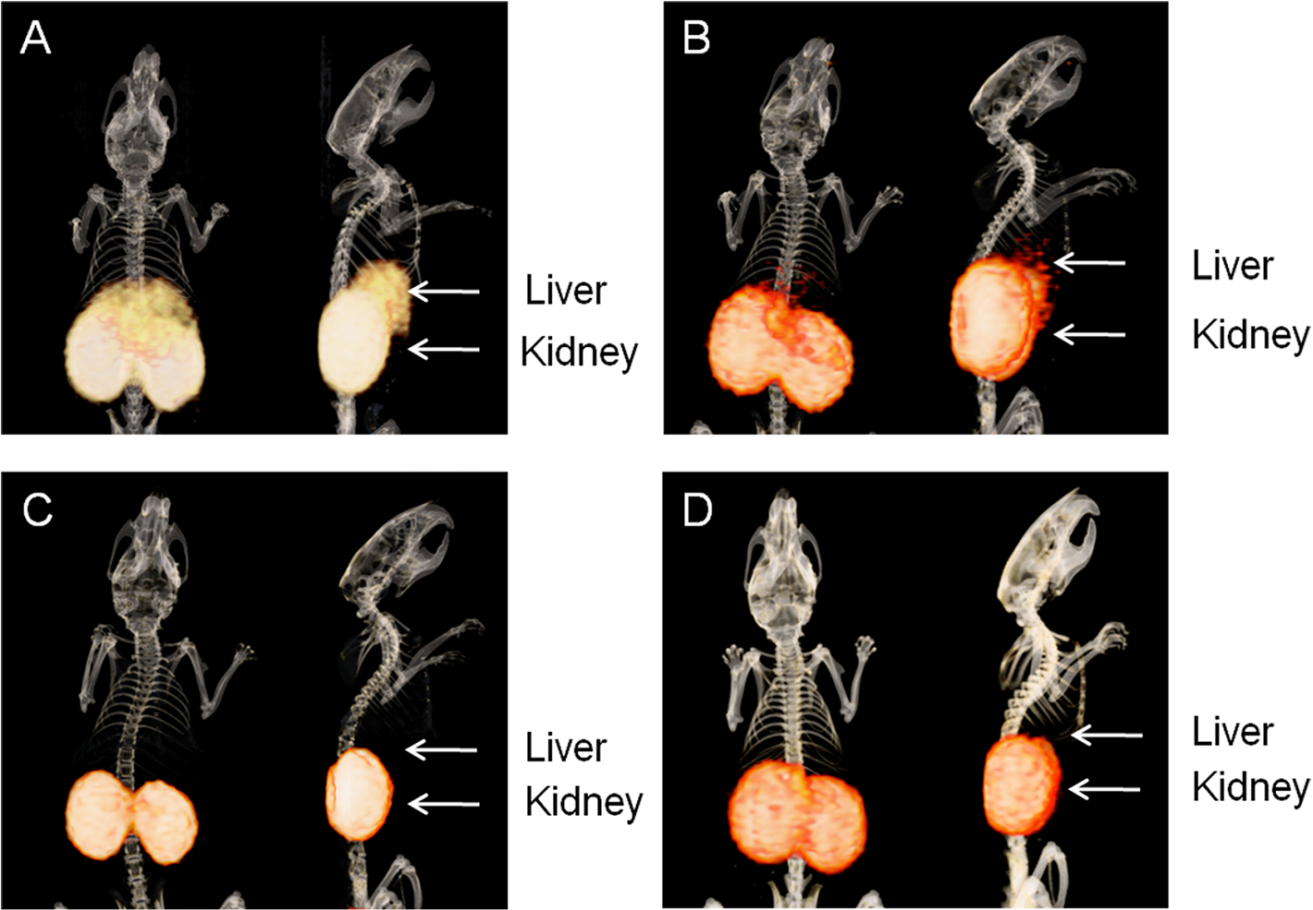
PET images of BALB/c nude mice after injection of 3 MBq ^68^Ga-DOTA-exendin-3. PET was acquired 1 h p.i. after euthanasia with an acquisition time of 45 min. High liver uptake is seen when the unpurified tracer is injected (**a**). Gel filtration partially removed the ^68^Ga-colloid (**b**), whereas no liver uptake was observed when ^68^Ga-DOTA-exendin-3 was purified by RP-HPLC (**c**) or SPE (**d**)

PET images of BALB/c nude mice after injection of unpurified, gel filtration, RP-HPLC-, or SPE-purified ^68^Ga-DOTA-exendin-3 are shown in Fig. 3. With unpurified ^68^Ga-DOTA-exendin-3, the liver was clearly visualized in the PET images (Fig. 3a). Liver visualization was less pronounced with ^68^Ga-DOTA-exendin-3 purified by gel filtration (Fig. 3b). No liver uptake was visible when ^68^Ga-DOTA-exendin-3 was purified with RP-HPLC (Fig. 3c) or SPE (Fig. 3d).

## Discussion

During the radiolabeling procedure of DOTA-conjugated compounds with ^68^Ga, in most cases, insoluble colloidal ^68^Ga species are formed, especially when compounds are labeled at a high specific activity. This insoluble ^68^Ga-colloid results in enhanced accumulation in the liver and spleen when injected in laboratory animals or patients, resulting in reduced image quality. Therefore, prevention of formation or removal of ^68^Ga-colloid is required. We evaluated three purification methods for the removal of ^68^Ga-colloid: solid phase extraction (SPE), preparative HPLC, and gel filtration. Solid phase extraction was a fast and simple method that effectively removed ^68^Ga-colloid from a labeling mixture of ^68^Ga-DOTA-exendin-3, resulting in negligible liver and spleen accumulation similar to that of ^111^In-DOTA-exendin-3. The major advantage of this technique is that purification of the labeling mixture can be performed within 5 min. Purification by preparative RP-HPLC resulted in similar spleen and liver accumulation. However, this technique is more time-consuming and less suited for purification of ^68^Ga-labeled tracers, due to the short half-life of ^68^Ga. Remarkably, the uptake of RP-HLPC-purified ^68^Ga-DOTA-exendin-3 in the pancreas was significantly higher. This is probably due to a slightly lower peptide dose, which results in higher pancreatic uptake as previously described [3]. Gel filtration removed ^68^Ga-colloid less efficiently, resulting in higher liver and spleen uptake than SPE and RP-HPLC purification. It should be noted that gel filtration could be more appropriate for the purification of larger compounds such as proteins. The higher molecular weight of proteins combined with the low molecular weight of ^68^Ga-colloid could result in better separation. However, the feasibility to separate ^68^Ga-colloid from high molecular weight compounds by gel filtration should be evaluated.

High specific activities of ^68^Ga-labeled tracers are required to administer high activity doses in combination with low peptide doses. It was shown that high peptide doses lead to reduced uptake in the target tissues due to (partly) saturation of the receptors [2–5], which is of particular importance in preclinical imaging. Moreover, high peptide doses can lead to (toxic) side effects, especially when biologically active compounds are used as the ligand. The need for a high specific activity generally enhances ^68^Ga-colloid formation, since incorporation of ^68^Ga present in the labeling mixture in the presence of low amounts of metal binding ligand might be incomplete. Since ^68^Ga(OH)_3_ could be formed at pH 3 in the absence of chelating agents (such as DOTA, DTPA, and EDTA) [11], ^68^Ga(OH)_3_ is formed in a labeling mixture at a pH between 3.5 and 4 when ^68^Ga-incorporation is incomplete. Interestingly, in the pH range generally used for ^68^Ga-labeling procedures (pH 3.5–4), Ga(III) is predominantly present as polymeric Ga(III) hydroxides, while Ga(OH)_3_ is the predominant form at pH 7 [12]. Furthermore, at higher pH, the formation of insoluble GaO(OH) can occur [13]. GaO(OH) is particularly formed at high temperature [14], which is of importance since most ^68^Ga-labeling procedures are carried out at high temperatures (80–100 °C). In contrast to Ga(III) hydroxides, GaO(OH) is only slowly redissolved even at low pH [14].

Wild et al. showed a similar biodistribution of ^68^Ga-labeled DOTA-exendin-4 [15] as ^68^Ga-DOTA-exendin-3 in our study. In the former study, no purification was performed of the ^68^Ga-labeled compound, and this lead to slightly higher uptake in the spleen as compared to the SPE and RP-HPLC purified tracer used in our study. However, the splenic uptake of ^68^Ga-DOTA-exendin-4 was lower than that of the unpurified ^68^Ga-DOTA-exendin-3 in our study. This lower uptake in the spleen of ^68^Ga-DOTA-exendin-4 reported by Wild et al. is probably due to a lower ^68^Ga-colloid content in the labeling mixture. The lower ^68^Ga-colloid content might be due to a lower specific activity of ^68^Ga-DOTA-exendin-4 and a different labeling protocol. In the study performed by Wild et al., the ^68^Ga-eluate was purified and the labeling was performed in a microwave for 5 min. The shorter labeling time in combination with the lower specific activity may lead to faster and more complete incorporation of ^68^Ga, reducing the risk of ^68^Ga-colloid formation [11]. Several other labeling methods [16, 17] and chelators [18–21] for labeling compounds with ^68^Ga are described with more efficient labeling yields and faster labeling kinetics. These new strategies for ^68^Ga-labeling of compounds might also reduce the risk of ^68^Ga-colloid formation.

We validated a TLC method for the detection of ^68^Ga-colloid in labeling mixtures of ^68^Ga-DOTA-exendin-3 and showed a clear linear correlation between added ^68^Ga-colloid to the labeling mixtures (free of ^68^Ga-colloid) and the measured ^68^Ga-colloid content. These results suggest accurate quantification of ^68^Ga-colloid in the labeling mixture. Determination of the colloid content is of great importance to calculate the specific activity. Neglecting the presence of ^68^Ga-colloid might result in overestimation of the specific activity and thus administration of a higher peptide dose as initially planned. This is especially true for exendin, where very low peptide doses need to be administered to prevent receptor saturation resulting in lower uptake in GLP-1R-positive tissues [3]. The detection limit of this method is approximately 3 % making detecting of very low concentrations of colloid difficult. The rather low sensitivity is due to tailing of the radiolabeling peptide hampering the clear delineation between the peak representing the ^68^Ga-colloid and the peak representing ^68^Ga-DOTA-exendin-3. The sensitivity of this method for the determination of ^68^Ga-colloid for other peptides might be different due to different migratory characteristics of the ^68^Ga-peptide on this TLC system compared to exendin.

Enhanced liver and spleen uptake will hamper the detection of lesions in the upper abdomen. Radiolabeled exendin was successfully used for detection of insulinomas [22] and could potentially be used for detection of the pancreatic beta cell mass. Enhanced liver and spleen uptake might reduce the sensitivity of these methods. Previous studies explored the feasibility of tumor imaging and atherosclerotic plaques with ^68^GaCl_3_ in mouse models for pancreatic adenocarcinoma and artherosclerosis, respectively [23, 24]. Although the tumor and atherosclerotic plaques could be detected, the PET images suffered from high background signal in the liver and lungs as well as high concentration in the blood. The binding of ^68^Ga to transferrin (prolonging the circulation time) and the formation of ^68^Ga-colloid could explain the enhanced background and is in line with our study.

## Conclusions

Solid phase extraction using a HLB cartridge is a fast and simple method to remove ^68^Ga-colloid. The uptake in the liver and spleen of the SPE-purified product was similar to that of ^111^In-DOTA-exendin-3 or HPLC-purified ^68^Ga-DOTA-exendin-3, indicating sufficient removal of insoluble ^68^Ga-colloid. Gel filtration only partly removed ^68^Ga-colloid species and is not suitable for purification of ^68^Ga-labeled exendin-3 and most likely other peptides. Moreover, SPE cartridges are routinely integrated in GMP-grade synthesis modules for ^68^Ga-labeling of peptide, and therefore, this method can be used for purification of ^68^Ga-labeled compounds for human use. Possibly, gel filtration might be suitable for larger compounds (e.g., antibodies, proteins), since the performance of size exclusion chromatography is expected to be superior for larger compounds. However, the feasibility for the removal of ^68^Ga-colloid by gel filtration of proteins and compounds where solid phase extraction is not possible should be verified. The studies presented here show the importance of complete removal of ^68^Ga-colloid before in vivo use.

## References

[1] Gabriel M, Decristoforo C, Kendler D, Dobrozemsky G, Heute D, Uprimny C (2007). 68Ga-DOTA-Tyr3-octreotide PET in neuroendocrine tumors: comparison with somatostatin receptor scintigraphy and CT. J Nucl Med.

[2] Breeman WA, de Jong M, Kwekkeboom DJ, Valkema R, Bakker WH, Kooij PP (2001). Somatostatin receptor-mediated imaging and therapy: basic science, current knowledge, limitations and future perspectives. Eur J Nucl Med.

[3] Brom M, Oyen WJ, Joosten L, Gotthardt M, Boerman OC (2010). 68Ga-labelled exendin-3, a new agent for the detection of insulinomas with PET. Eur J Nucl Med Mol Imaging.

[4] Froidevaux S, Calame-Christe M, Schuhmacher J, Tanner H, Saffrich R, Henze M (2004). A gallium-labeled DOTA-alpha-melanocyte-stimulating hormone analog for PET imaging of melanoma metastases. J Nucl Med.

[5] Notni J, Steiger K, Hoffmann F, Reich D, Kessler H, Schwaiger M, et al. Variation of specific activities of Ga-68-Aquibeprin and Ga-68-Avebetrin enables selective PET-imaging of different expression levels of integrins alpha5beta1 and alphavbeta3*.* J Nucl Med 2016. doi:10.2967/jnumed.116.173948. 10.2967/jnumed.116.173948 27151985

[6] Antunes P, Ginj M, Zhang H, Waser B, Baum RP, Reubi JC (2007). Are radiogallium-labelled DOTA-conjugated somatostatin analogues superior to those labelled with other radiometals?. Eur J Nucl Med Mol Imaging.

[7] Breeman WA, de Jong M, de Blois E, Bernard BF, Konijnenberg M, Krenning EP (2005). Radiolabelling DOTA-peptides with 68Ga. Eur J Nucl Med Mol Imaging.

[8] Sandstrom M, Velikyan I, Garske-Roman U, Sorensen J, Eriksson B, Granberg D (2013). Comparative biodistribution and radiation dosimetry of 68Ga-DOTATOC and 68Ga-DOTATATE in patients with neuroendocrine tumors. J Nucl Med.

[9] Velikyan I, Sundin A, Sorensen J, Lubberink M, Sandstrom M, Garske-Roman U (2014). Quantitative and qualitative intrapatient comparison of 68Ga-DOTATOC and 68Ga-DOTATATE: net uptake rate for accurate quantification. J Nucl Med.

[10] Sosabowski JK, Mather SJ (2006). Conjugation of DOTA-like chelating agents to peptides and radiolabeling with trivalent metallic isotopes. Nat Protoc.

[11] Green MA, Welch MJ (1989). Gallium radiopharmaceutical chemistry. Int J Rad Appl Instrum.

[12] Hacht B (2008). Gallium(III) ion hydrolysis under physiological conditions. B Korean Chem Soc.

[13] Gamsjage H, Schindle P (1967). Loslichkeitsprodukte Von Metalloxiden Und -Hydroxiden .11. Die Loslichkeit Von Alpha-Gao(Oh) Bei 60 Degrees C in Perchlorsauren Losungen Konstanter Ionenstarke. Helv Chim Acta.

[14] Uchida M, Okuwaki A (1998). Potentiometric determination of the first hydrolysis constant of gallium(III) in NaCl solution to 100 degrees C. J Solution Chem.

[15] Wild D, Wicki A, Mansi R, Behe M, Keil B, Bernhardt P (2010). Exendin-4-based radiopharmaceuticals for glucagonlike peptide-1 receptor PET/CT and SPECT/CT. J Nucl Med.

[16] Schultz MK, Mueller D, Baum RP, Leonard Watkins G, Breeman WA (2013). A new automated NaCl based robust method for routine production of gallium-68 labeled peptides. Appl Radiat Isot.

[17] Shetty D, Jeong JM, Ju CH, Kim YJ, Lee JY, Lee YS (2010). Synthesis and evaluation of macrocyclic amino acid derivatives for tumor imaging by gallium-68 positron emission tomography. Bioorg Med Chem.

[18] Eder M, Wangler B, Knackmuss S, LeGall F, Little M, Haberkorn U (2008). Tetrafluorophenolate of HBED-CC: a versatile conjugation agent for 68Ga-labeled small recombinant antibodies. Eur J Nucl Med Mol Imaging.

[19] Ma MT, Neels OC, Denoyer D, Roselt P, Karas JA, Scanlon DB (2011). Gallium-68 complex of a macrobicyclic cage amine chelator tethered to two integrin-targeting peptides for diagnostic tumor imaging. Bioconjug Chem.

[20] Notni J, Pohle K, Wester HJ (2012). Comparative gallium-68 labeling of TRAP-, NOTA-, and DOTA-peptides: practical consequences for the future of gallium-68-PET. EJNMMI Res.

[21] Notni J, Simecek J, Hermann P, Wester HJ (2011). TRAP, a powerful and versatile framework for gallium-68 radiopharmaceuticals. Chemistry.

[22] Wild D, Macke H, Christ E, Gloor B, Reubi JC (2008). Glucagon-like peptide 1-receptor scans to localize occult insulinomas. N Engl J Med.

[23] Silvola JMU, Laitinen I, Sipila HJ, Laine VJO, Leppanen P, Yla-Herttuala S (2011). Uptake of (68)gallium in atherosclerotic plaques in LDLR(−/−)ApoB(100/100) mice. EJNMMI Res.

[24] Ujula T, Salomaki S, Autio A, Luoto P, Tolvanen T, Lehikoinen P (2010). Ga-68-chloride PET reveals human pancreatic adenocarcinoma xenografts in rats-comparison with FDG. Mol Imaging Biol.

